# Co-composting of cattle manure with biochar and elemental sulphur and its effects on manure quality, plant biomass and microbiological characteristics of post-harvest soil

**DOI:** 10.3389/fpls.2022.1004879

**Published:** 2022-09-29

**Authors:** Jiri Holatko, Tereza Hammerschmiedt, Antonin Kintl, Adnan Mustafa, Muhammad Naveed, Tivadar Baltazar, Oldrich Latal, Petr Skarpa, Pavel Ryant, Martin Brtnicky

**Affiliations:** ^1^ Department of Agrochemistry, Soil Science, Microbiology and Plant Nutrition, Faculty of AgriSciences, Mendel University in Brno, Brno, Czechia; ^2^ Agrovyzkum Rapotin, Ltd., Rapotin, Czechia; ^3^ Agricultural Research, Ltd., Troubsko, Czechia; ^4^ Institute of Chemistry and Technology of Environmental Protection, Faculty of Chemistry, Brno University of Technology, Brno, Czechia; ^5^ Institute for Environmental Studies, Faculty of Science, Charles University in Prague, Praha, Czechia; ^6^ Institute of Soil and Environmental Science, University of Agriculture Faisalabad, Faisalabad, Pakistan

**Keywords:** manure enrichment, soil nutrients, organic matter, soil amendments, fertilizers, modified biochar

## Abstract

Improvement of manure by co-composting with other materials is beneficial to the quality of the amended soil. Therefore, the manure was supplied with either biochar, elemental sulphur or both prior to fermentation in 50 L barrels for a period of eight weeks. The manure products were subsequently analyzed and used as fertilizers in a short-term pot experiment with barley fodder (*Hordeum vulgare* L.). The experiment was carried out under controlled conditions in a growth chamber for 12 weeks. The sulphur-enriched manure showed the lowest manure pH and highest ammonium content. The co-fermentation of biochar and sulphur led to the highest sulphur content and an abundance of ammonium-oxidizing bacteria in manure. The biochar+sulphur-enriched manure led to the highest dry aboveground plant biomass in the amended soil, whose value was 98% higher compared to the unamended control, 38% higher compared to the variant with biochar-enriched manure and 23% higher compared to the manure-amended variant. Amendment of the sulphur-enriched manure types led to the highest enzyme activities and soil respirations (basal, substrate-induced). This innovative approach to improve the quality of organic fertilizers utilizes treated agricultural waste (biochar) and a biotechnological residual product (elementary sulphur from biogas desulphurization) and hence contributes to the circular economy.

## 1 Introduction

Widely observed soil degradation is currently one of the main global concerns. It is caused by a combination of natural and anthropogenic detrimental processes, such as pollution, deforestation and consequences of poor land management and unsustainable agricultural practices: wind and water erosion, physicochemical changes as compaction, salinization, acidification, loss of soil organic matter (SOM) and nutrients (Virto et al., 2014; Tetteh, 2015; EC, European Commission, 2021). Additionally, agriculture intensification, driven by a necessity to meet human needs, has resulted in serious threats of soil pollution, environmental degradation and climate change (Lal, 2020).

The loss of SOM is closely related to the decline in soil fertility and the biological function of soils (Lal, 2009). Application of organic fertilizers in this regard can restore and preserve the sustainable SOM content in soil (Liu et al., 2011). Farmyard manure is the most common type of organic fertilizer. It plays a significant role in maintaining high quality healthy arable soils and sustainable agriculture (Kirchmann and Thorvaldsson, 2000). According to the recent literature, manure application to agricultural soil has a positive effect on the build-up of SOM and thus improves the soil structure as well as the intrinsic fertility of the soils (Mustafa et al., 2020; Mustafa et al., 2021). In addition, manure application may significantly increase the soil water storage and crop yield and has a positive effect on soil microbial activity (Wang et al., 2016; Hoover et al., 2019; Ashraf et al., 2021). It represents a good source of nutrients, especially carbon (C), nitrogen (N), phosphorus (P) and minerals, for both plants and soil organisms, including microbes (Qaswar et al., 2019). However, the properties of manure are variable and depend mainly on the type of livestock, bedding material and the conditions of fermentation, which can be modified to achieve the intended quality of product (Naveed et al., 2021).

Therefore, amendment of soil with manure as the primary source of organic matter, enriched with biochar and elemental sulphur (S), has brought promising results in previous studies. Fermentation of biochar-enriched manure mitigated emissions of greenhouse gases (Rogovska et al., 2011; Maurer et al., 2017), ammonia (Janczak et al., 2017), prevented nutrient losses (Hagemann et al., 2018). Biochar addition modified the thermodynamics and heat generation in the fermentation process (Czekała et al., 2016) and changed the content and functional diversity of microorganisms (He et al., 2018), as well as microbial mineralization (Jindo et al., 2012) in manure.

Moreover, the effect of S on manure composting is the subject of several studies in the recent literature. Sulphur is not only a useful nutrient for microorganisms and plants (Skwierawska et al., 2016; Bouranis et al., 2019), but also serves as soil conditioner improving the physicochemical properties of soil (Skwierawska et al., 2008; Abou Hussien et al., 2020). It has also been shown to increase crop yields (Soltanaeva et al., 2018). The S deficiency in Europe’s agricultural soils is linked to a significant decline in sulphur dioxide (SO_2_) emissions, which have been reduced by 70–80% over the last 30 years (Hoesly et al., 2018). For example, the available results of soil analyses carried out in the Czech Republic show that 85% of samples have low S content (Kulhánek et al., 2018). The effect of elemental S (upon the combined treatments with manure or biochar) on manure quality, soil properties and plant growth has been reported in only a few studies, e.g. (Mahimairaja et al., 1994b; de la Fuente et al., 2007; Godlewska, 2018b), and thus has left room for further studies. Moreover, the amendment of co-composted manure with elemental S may significantly alter soil enzyme activity (Malik et al., 2021), with a putative benefit of increased rate of nutrient transformation *via* enhancement of microbial activity and abundance by combination of external organic matter and elemental S amendment (Hammerschmiedt et al., 2021; Malik et al., 2021). Biochar addition to soil was also referred to affect activity of nutrient-transforming enzymes in soil not only negatively (Li et al., 2018; Song et al., 2019), but also positively (Azeem et al., 2019; Zhang et al., 2021). The novelty of this research lies in the pre-maturation enrichment of manure with elemental S, which is assumed to be promoted during the manure fermentation to the accelerated transformation into plant-available form and modulated in this process by a presence of biochar.

The objectives of this study were to evaluate (I) the impact of manure enrichment (prior to fermentation) with biochar, elemental S and a combination of both on the fertilizing properties of produced manure types, (II) the effect of soil amendment with these various manure types on the chemical and biological properties (i.e. activity of nutrient-transforming soil enzymes), and biomass of a test crop, barley fodder (*Hordeum vulgare* L.). It was hypothesized that the acidifying effect of elemental S would counteract the alkalizing effect of biochar in the case of their co-fermentation in manure, and that it could modify the biological properties of manure *via* S oxidation-promoted nutrient mineralization, accompanied by reduced ammonia emission.

## 2 Materials and methods

### 2.1 Collection, preparation, and analysis of modified manure

Animal manure was collected from a cattle-breeding farm of Research Institute for Cattle Breeding Ltd., located in the village of Rapotin, Czech Republic, Central Europe (49°58’46.4” N, 17°0’26,6” E). Experimental matured manure was prepared in the 50 L sealable containers (three containers per variant), filled with 10 kg of collected manure, which was (optionally) mixed with biochar and elemental S to create four experimental variants: [M] manure, [M+B] manure + biochar (40 g·kg^-1^), [M+S] manure + elemental S (1.4 g·kg^-1^), [M+B+S] manure + biochar (40 g·kg^-1^) + elemental S (1.4 g·kg^-1^). Each variant was prepared in three replicates. Used biochar was produced from agricultural waste at 600°C (Sonnenerde GmbH, Riedlingsdorf, Austria), and its properties were according the analyses of manufacturer as follows: elements (in g·kg^-1^) - C 866, N 3.0, O 10.0, H 14.2; Ash_550°C_ 11.7%, salts 0.42%, pH (CaCl_2_) 8.5. Elemental S was a waste product obtained during desulphurization of biogas at sugar factory biogas plant in THIOPAQ scrubber (Paques, Netherlands).

The activation process ran for eight weeks at a laboratory-controlled temperature (20 ± 2°C) at stable air humidity (measured weekly). At the end of the process, a mixed sample from each variant was taken and analyzed. Manure pH in CaCl_2_ was determined according to (ISO 10390:2005); total Kjeldahl nitrogen (TKN) was determined according to (ISO 11261:1995); and ammonium nitrogen (N-NH_4_) was measured according to (ISO 15476:2009). The available P was determined according to (Egnér et al., 1960); dry matter (DM) was measured gravimetrically (Hoskins et al., 2003); and organic C (C_org_) was measured according to (EN 15936:2012). Total S was determined according to (EN 15749:2009), ammonium-oxidizing bacteria (AOB) according to (Rotthauwe et al., 1997), denitrifying microorganisms (*nirS*) according to (Kandeler et al., 2006) and S-reducing microorganisms (*dsr*) according to (Ben-Dov et al., 2007).

### 2.2 Pot experiment

All four produced manure types were used as soil amendments in pot experiments with barley fodder (*Hordeum vulgare* L.) as a test crop. All experimental pots (volume 5 L) were filled with soil substrate: fine quartz sand (0.1–1.0 mm) mixed with sieved (2.0 mm) topsoil (0–15 cm) from the rural area near the town of Troubsko, Czech Republic - 49°10’28”N 16°29’32”E in ratio 1:1, w/w. The soil was a silty clay loam (according to USDA Textural Triangle), Haplic Luvisol [according to WRB soil classification (FAO, Food and Agriculture Organization of the United Nations, 2014)], and its properties were as follows: soil macronutrients (g·kg^-1^) - total C 14.00, total N 1.60 - available nutrients (mg·kg^-1^) - P 97, S 100, Ca 3259, Mg 236, K 231; mineral N forms (mg·kg^-1^) – N_min_ 62.84, N-NO_3_ 56.80, N-NH_4_ 6.04; pH (CaCl_2_) 7.3.

The four pot experimental variants were made by thoroughly mixing a soil:sand blend (5 kg) with the particular manure type in amounts of 200 g per pot (the manure amount being equal to 50 t·ha^-1^). An unamended control contained only 5 kg of soil:sand blend. The treatments included (1) control, (2) manure (M), (3) manure + biochar (M+B), (4) manure + elemental S (M+S), (5) manure + biochar + elemental S (M+B+S). Each variant was prepared in five replicates. Each pot was sown with 16 barley seeds 2 cm under the soil surface and was watered with distilled water to achieve 65% water-holding capacity (WHC). This moisture level was maintained throughout the entire experiment. All pots were placed randomly into a growth chamber (CLF Plant Climatics GmbH, Germany). Controlled conditions were set as follows: 12-hours photoperiod, light intensity 20 000 lx, temperature (day/night) 20/12°C, relative air humidity (day/night) 45%/70%. After 14 days, the number of plants was reduced to 12 in each pot. Moreover, the pots were randomly rotated every other day to ensure the homogeneity of the conditions for the treatments.

### 2.3 Plant biomass measurements

The barley plants were grown for 12 weeks. After that, the shoots were cut at the ground level, washed with distilled water (Iocoli et al., 2019), and dried at 60°C until a constant weight was obtained. The dry aboveground biomass (AGB) was determined gravimetrically using the analytical scales.

### 2.4 Post-harvest soil characterization and statistical analysis

The soil samples were taken after the harvesting of AGB of barley. The homogenization of the samples was done by sieving through a 2 mm mesh. The samples for the enzyme activity assays (ISO 20130:2018) – β-glucosidase (GLU), arylsulfatase (ARS), phosphatase (Phos), N-acetyl-β-D-glucosaminidase (NAG) and urease (Ure) – were freeze-dried. The samples stored at 4°C were used for determination of dehydrogenase activity (DHA), soil basal (BR) and substrate induced respirations (Campbell et al., 2003): D-glucose (Glc-SIR), D-trehalose (Tre-SIR), citric acid (Cit-SIR), N-acetyl-β-D-glucosamine (NAG-SIR), L-alanine (Ala-SIR), L-lysine (Lys-SIR) and L-arginine (Arg-SIR). The total soil carbon (TC) and nitrogen (TN) content (ISO 10694:1995, ISO 13878:1998) were analyzed using air-dried samples.

DHA was measured by 2,3,5-triphenyltetrazolium chloride (TTC)-based method. The *p*-nitrophenol (PNP)-derivatives of the specific soil substrates were used for Vis spectrophotometric measurement (Infinite M Nano, Tecan Trading AG, Switzerland) at Λ = 405 nm (β-glucosidase, arylsulfatase, phosphatase, and N-acetyl-β-D-glucosaminidase). Urease activity was determined as an amount of ammonium produced from the substrate urea, detected Vis spectrophotometrically by the reagent cyanurate (Λ = 650 nm). Other soil properties were determined by the standard methods and the data obtained was statistically analyzed as listed in (**Table 1**).

**Table 1:** Determined soil properties, methods used for measurement and statistics, relevant references.

Property	Method	Unit	Reference
Total soil carbon	Dry combustion using, LECO TruSpec analyzer (MI USA)	mg·g^-1^	(ISO 10694:1995)
Total soil nitrogen			(ISO 13878:1998)
Dehydrogenase activity	Triphenyl tetrazolium chloride (TTC)-based method	µg TPF·g^-1^·h^-1^	(Doi and Ranamukhaarachchi, 2009)
Soil enzyme activities (GLU, ARS, Phos, NAG, Urea)	Microplate incubation, Vis spectrophotometry	µmol PNP·g^-1^·h^-1^, µmol NH_3_·g^-1^·h^-1^	(ISO 20130:2018)
Basal soil respiration	MicroResp^®^ device	μg CO_2_·g^-1^·h^-1^	(Campbell et al., 2003)
Substrate induced soil respiration	MicroResp^®^ device + inducers (sugars, amino acids)		
**Processing**	**Method**	**Tool**	**Reference**
Statistical analysis	Multivariate analysis of variance (MANOVA), one-way analysis of variance (ANOVA) with Tukey’s *post-hoc* test, principal component analysis (PCA), Pearson’s correlation analysis	Program R version 3.6.1.	(Holatko et al., 2020)

The Shapiro–Wilk and the Levene tests (at p ≤ 0.05) were performed for the verification of normality and homogeneity of variances. Principal component analysis (PCA), and one-way analysis of variance (ANOVA) type I (sequential) sum of squares at 5% significance level were used for characterization of relationship between the treatments and selected soil properties. Tukey’s HSD (honestly significant difference) test was used for detection the statistically significant difference among factor level means, and “treatment contrast” was calculated as factor level means for each treatment. The results were also graphically presented with Rohlf biplot for standardized PCA. Pearson correlation analysis was performed for measuring the linear dependence between soil properties. Pearson correlation coefficient was interpreted as follows: 0.0< r< 0.3 (negligible correlation), 0.3< r< 0.5 (low correlation), 0.5< r< 0.7 (moderate correlation), 0.7< r< 0.9 (high correlation), and 0.9< r< 1.0 (very high correlation).

## 3 Results

### 3.1 Effect of added amendments on pH and nitrogen forms in manure

**Figure 1:**
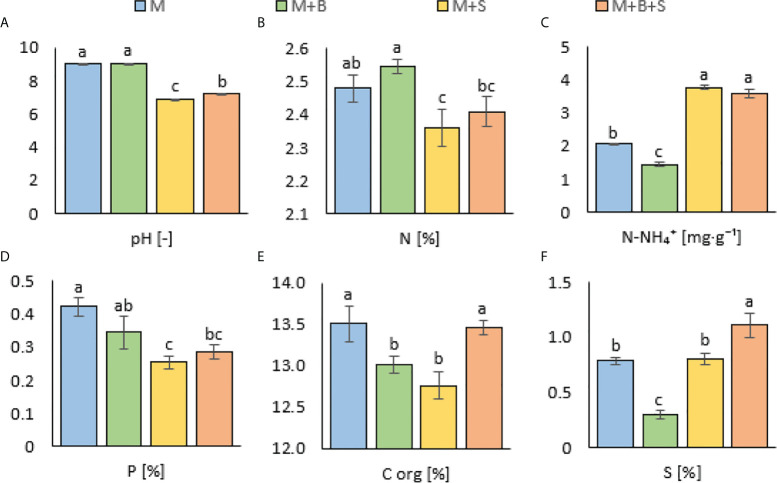
Properties of the maturated manures enriched with additives (biochar and S). **(A)** pH, **(B)** total Kjeldahl nitrogen, **(C)** ammonium nitrogen, **(D)** available phosphorus, **(E)** organic carbon, **(F)** total sulphur. Different letters indicate differences at level of significance p ≤ 0.05.

It was observed that both M+S and M+B+S exerted significantly lower pH values (6.85 ± 0.01 and 7.21 ± 0.01, respectively) compared to the M and M+B (9.04 ± 0.01 and 9.05 ± 0.01, respectively) – (**Figure 1A**). The S enrichment of the M+S variant caused a significantly lower TKN (by 4.8%) value but a significantly higher N-NH_4_ (by 83.1% as compared to the M) – (**Figures 1B, C**). The M+S manure did not differ in both TKN and N-NH_4_ content from the M+S+B manure, whereas this variant showed significantly decreased total TKN (by 7.2%) and increased N-NH_4_ (by 161%) content compared to the M+B.

### 3.2 Effect of added amendments on manure – derived phosphorus and organic carbon

In both S-enriched variants (M+S and M+B+S), the available P was decreased compared to the M (by 39% and 32%, respectively) – (**Figure 1D**). The M+B variant showed significantly lower C_org_ compared to the M (by 3.7%) and M+B+S. A similar decrease in the C_org_ content was detected in the M+S variant (by 5.5% compared to M) – (**Figure 1E**).

### 3.3 Effect of added materials on sulphur content in manure

**Figure 2:**
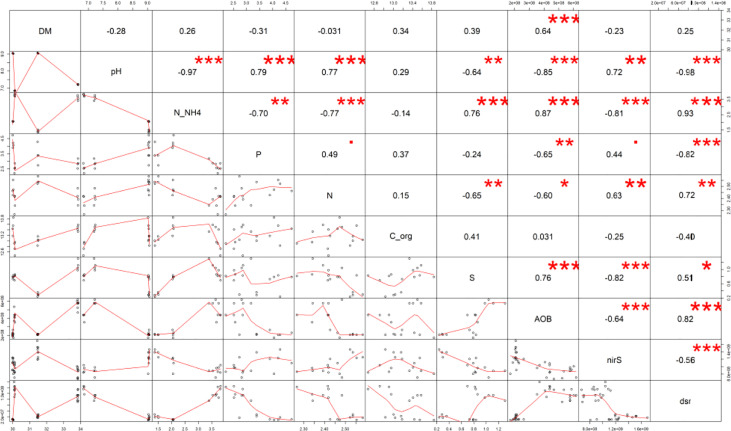
The Pearson’s correlation matrix of the maturated manure properties. Explanation: Significance at · p ≤ 0.10; *p ≤ 0.05; **p ≤ 0.01; ***p ≤ 0.001.

**Figure 3:**
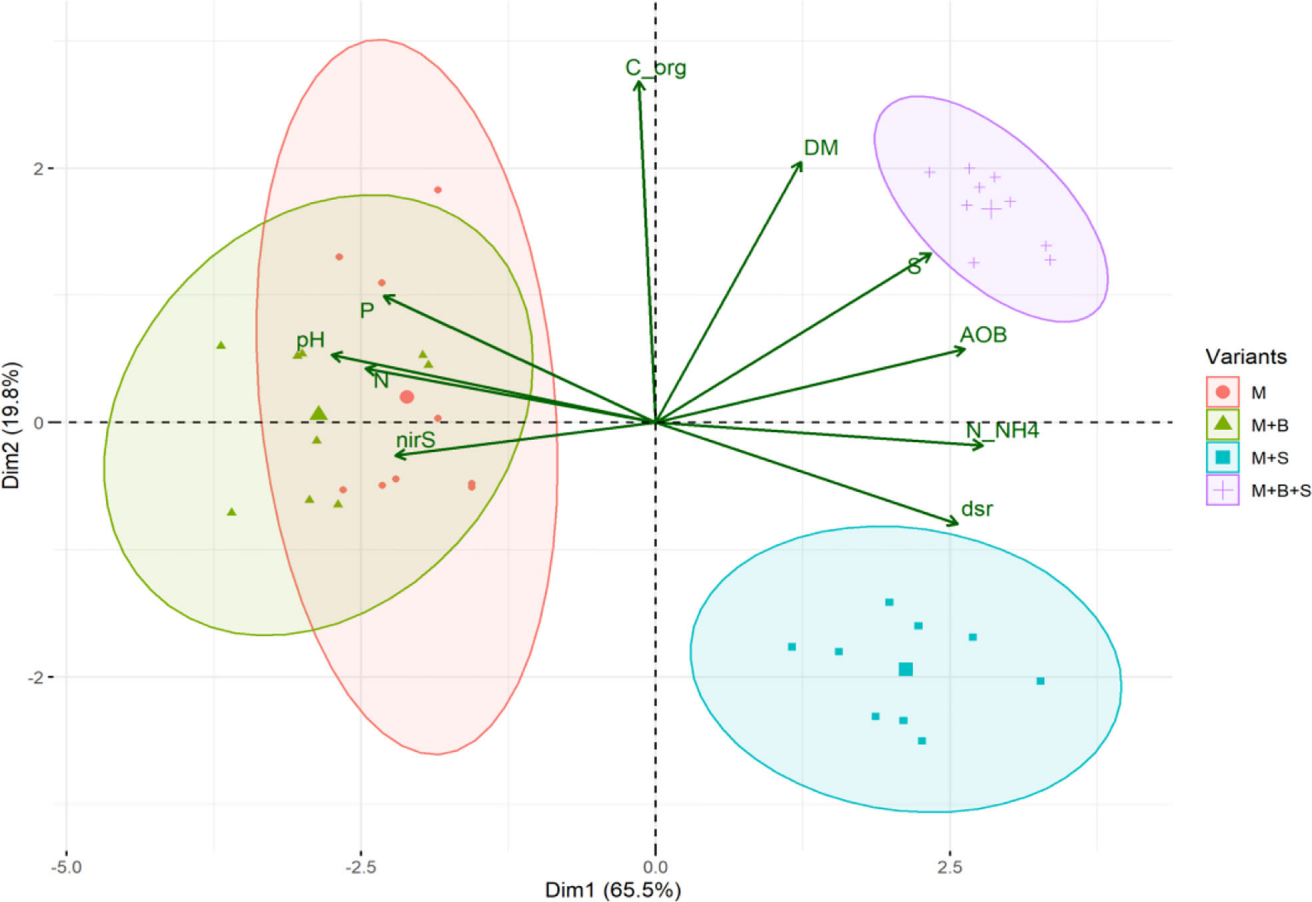
The PCA biplot of the maturated manure properties.

A significantly decreased total S value for the M+B manure (by 62% compared to the M) was received, whereas the M+B+S variant was significantly the highest (41% higher than M) – (**Figure 1F**). The total S in manure was significantly related to the microbiological traits of *dsr* (p ≤ 0.05, r = 0.51), and (at p ≤ 0.00) AOB (r = 0.76), *nirS* (r = -0.82), and to the N-NH_4_ nitrogen (r = 0.76) (**Figure 2**). These relationships are apparent also from the PCA biplot (**Figure 3**).

### 3.4 Effect of added amendments on microbial abundance in manure

**Figure 4:**
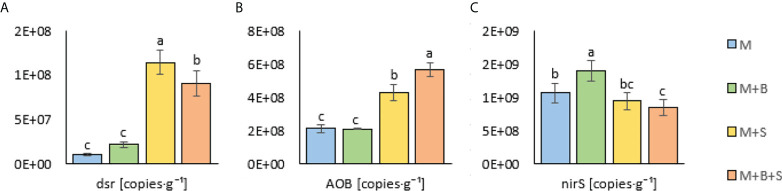
Microbial properties of the maturated manures enriched with additives (biochar and S). **(A)** sulphur-reducing, **(B)** ammonia-oxidizing, and **(C)** denitrifying microorganisms in the maturated manures enriched with additives. Different letters indicate differences at level of significance p ≤ 0.05.

Significantly increased *dsr* (determinant of the S-reducing microorganisms) was found the in the M+S and M+B+S variants (supplied with S) compared to the M and M+B (non-supplied with S): the values were ∼10-fold and ∼7.7-fold higher (than M), respectively (**Figure 4A**). The addition of S to the unmatured manure was crucial for the abundance of N-transforming microbiota: *dsr* correlated positively (p ≤ 0.001) with AOB (r = 0.82) and N-NH_4_ (r = 0.93), whereas *nirS* correlated negatively (p ≤ 0.001) with N-NH_4_ (r = -0.81), S (r = -0.82), AOB (r = -0.64) and *dsr* (r = -0.56) (**Figure 2**).

The AOB was significantly increased in the M+S and M+B+S variants (by 102% and 169%) compared to the M and M+B; the significantly highest AOB value was detected in the M+B+S manure (**Figure 4B**). The significantly lowest nirS value was revealed in the M+B+S variant (20.5% lower than M) and the significantly highest in the M+B variant (27.4% higher than M) (**Figure 4C**)

### 3.5 Effect of manure types on soil fertility and plant biomass yield

**Figure 5:**
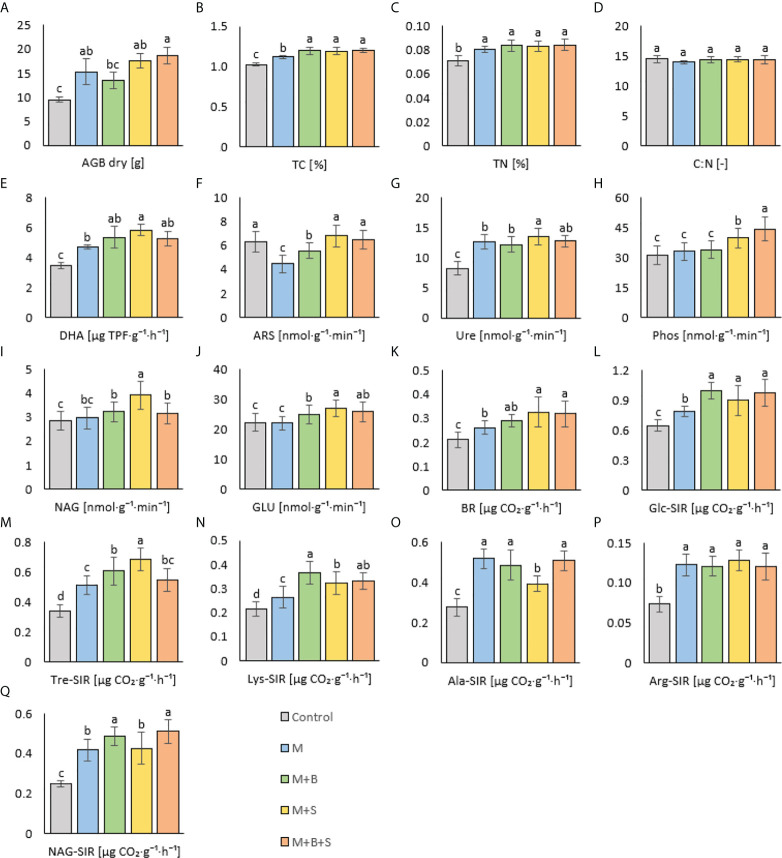
Dry plant above ground biomass and soil properties of variants amended with various manure types. **(A)** dry above ground biomass, **(B)** total carbon, **(C)** total nitrogen, **(D)** C:N ratio, **(E)** dehydrogenase activity, **(F)** arylsulfatase act., **(G)** urease act., **(H)** phosphatase act., **(I)** N-acetyl-β-D-glucosaminidase act., **(J)** β-glucosidase act., **(K)** basal respiration, **(L)** D-glucose-induced resp., **(M)** D-trehalose-induced resp., **(N)** L-lysine-induced resp., **(O)** L-alanine-induced resp., **(P)** L-arginine-induced resp., **(Q)** N-acetyl-β-D-glucosamine-induced resp. Different letters indicate differences at level of significance p ≤ 0.05.

All manure-amended variants (M, M+B, M+S, M+B+S) showed a significant increase (by 62%, 43%, 86%, 98%, respectively) of AGB compared to the AGB of the control (**Figure 5A**). Further, the AGB value of the variant M+B+S was significantly higher than the AGB of M+B. The positive significant correlation (p ≤ 0.001, r = 0.69) that was found for AGB and TC, BR, Ure, corroborated the relation between plant biomass and soil nutrient availability. These values were significantly increased in M+B+S soil compared to the control soil (Ure) as well as compared to both the control and M variant (TC, BR).

The TC and TN content in the amended soil variants (M, M+B, M+S, and M+B+S) was significantly higher compared to the control: by 9%, 17%, 16%, 18% (TC) and by 13%, 18%, 17%, 19% (TN) (**Figures 5B, C**). TN values were similar among all these manure-supplied variants, whereas the TC was significantly lower in the M variant compared to the M+B, M+S and M+B+S. The variants did not differ significantly in C:N ratio (**Figure 5D**).

### 3.6 Effect of manure types on soil microbial activity

Significantly increased DHA was reached in all manure-amended variants (M, M+B, M+S, M+B+S) compared to the control: the values were higher by 35%, 53%, 67%, 50% (**Figure 5E**). Furthermore, the significantly higher DHA value was obtained in the M+S variant compared to the M variant. It assumed a general effect of M+S amendment on the microbial soil activity because DHA correlated significantly (p ≤ 0.001) positively with Ure (r = 0.79), BR (r = 0.54) and GLU (r = 0.53). The values of ARS in the soil variants amended with S-enriched manures (M+S, M+B+S) were not significantly higher than in the control soil (**Figure 5F**), whereas the variants M and M+B showed significantly lower ARS values (by 29% and 12%, respectively). Nevertheless, the amendment of S-enriched manures to soil resulted in a demonstrated significant increase (in M+S and M+B+S, compared to the control) of Ure (by 64% and 55%, **Figure 5G**), Phos (by 28% and 42%, **Figure 5H**), NAG (by 37% and 11%, **Figure 5I**) and GLU (by 21% and 16%, **Figure 5J**) activities. The values in M+S were significantly higher for Ure, NAG and GLU compared to the M and M+B values. The Phos was highest in M+B+S.

Compared to the control, BR was significantly increased in variants amended with all types of manure. Moreover, the received BR values were significantly higher in the S-enriched manure-treated variants (M+S and M+B+S, by 54% and 51% compared to control) than in the non-enriched manure variant M (by 24% higher than control) – (**Figure 5K**).

Results similar to the BR determination were obtained for substrate induced respirations, Glc-SIR, Tre-SIR, Lys-SIR; the control soil exerted significantly lower respiration values compared to the amended soil variants, and these showed significantly higher values due to the addition of enriched manures (M+B, M+S, M+MB+S) than after the addition of the control sole manure M (**Figures 5L-N**). On the contrary, the Ala-SIR (**Figure 5O**) and Arg-SIR (**Figure 5P**) showed in variants M+B and M+B+S no difference to the M variant, whereas the Ala-SIR in M+S was lower compared to the M variant. NAG-SIR was significantly decreased in non-biochar-amended variants (M and M+S) compared to the biochar-treated variants (M+B and M+B+S) (**Figure 5Q**).

## 4 Discussion

### 4.1 Effect of added amendments on pH and nitrogen forms in manure

The lower pH of the M+S and M+B+S variants (compared to the M and M+B) was ascribed to the acidifying potential of the elemental S addition, which was already reported (de la Fuente et al., 2007). Such biological oxidation of elemental S added to the alkaline mixed manure was referred to by (Costello et al., 2019). On the contrary, biochar in the manure M+B caused no pH change compared to the unamended manure (M), probably due to a negligible difference in the pH of the blended materials (manure and biochar). A significantly higher pH in M+B+S compared to the M+S variant was presumably caused by the neutralizing effect of added biochar, due to the sorption of S on its surface, such as reported by (Xu et al., 2014). These findings corroborated our hypotheses. The pH effect on other manure properties was ascribed from pH-significant (p ≤ 0.001) correlations: positive with TKN (r = 0.77), P (r = 0.79), *nirS* (r = 0.72), and negative with N-NH_4_ (r = -0.97), AOB (r = -0.85), *dsr* (r = -0.98) – these relations are also apparent from the PCA biplot.

Whereas the M+S variant showed significantly decreased TKN but significantly increased ammonium nitrogen compared to the unenriched manure; the M+S+B manure exerted significantly increased N-NH_4_ content and decreased TKN compared to the M+B. The S-enriched variants (M+S, M+B+S) showed higher ammonium content than non-S-enriched ones, putatively due to increased acidity, which was coupled with the microbial production of H_2_SO_4_. Sulphuric acid may promote activity of proteolytic bacteria, neutralize and protonate NH_3_ and, thus, mitigate its release from the manure. This mechanism is in line with the findings of (Mahimairaja et al., 1994b). Moreover, nitrification activity has a pH optimum for oxygen uptake between 7.0 and 7.4. Despite the presumed reduction in N loss *via* volatilization with S-treatment of the manure, higher TKN content in M and M+B (as compared to M+S and M+B+S) was observed. Concurrent with the previously reported benefit of acid manure to nitrification, an acidic pH increased the formation of bicarbonate during the hydrolysis of uric acid and urea (Vlek and Stumpe, 1978). Bicarbonate in manure may (opposite to the effect of sulphuric acid) cause higher losses of NH_3_. The reduction in nitrogen losses due to its immobilization during co-composting with carbonaceous biochar-derived materials, referred to by (Wang et al., 2018; Nguyen et al., 2022), could also be involved.

### 4.2 Effect of added amendments on manure – derived phosphorus and organic carbon

The availability of P in the manure variants seemed to be pH-dependent and significantly related to the S reduction (r = -0.82, p ≤ 0.001) and ammonium content (r = -0.70, p ≤ 0.01). Both M+S and M+B+S exerted the available P content lower than the manure M. These results may be explained by the acidifying effect of either sulphuric acid (H_2_SO_4_) or hydrogen sulphide (H_2_S) and increased access of protons from acidified ammonium $\left(NH_{4}^{+}\right)$ , all of which factors favored the precipitation of P. Previous studies (Mahimairaja et al., 1994a; Penn and Camberato, 2019) referred to these mechanisms, which make phosphates less soluble at a low pH. The single-enriched variants (M+B, M+S) showed significantly lower C_org_ compared to the M and M+B+S. The TC content was close to the TN content. The access of biochar carbon putatively affected the C_org_ content in the variants M+B (and also M+B+S), which showed highest decomposition and C mineralization level. The highest composting rate could lead to increased C volatilization in the form of CO_2_ (or CH_4_) (Jiang et al., 2011). However, no excessive C_org_ source was added to the unmatured manure of this variant. Elemental S was presumed to increase microbial abundance and stimulate the microbial decomposing activity (Roig et al., 2004). Moreover, elemental S may enhance the formation of sulphuric acid, as was described by de la Fuente et al. (2007). The authors of the study revealed that sulphuric acid combined with carbonate materials leads to the production of sulphates and the removal of carbonates (in the form of CO_2_). A significantly higher microbial activity in the M+B+S variant was presumed too and related to the evidence of increased aeration, which may cause desiccation similar as reported severe drying in the compost (Sundberg, 2005).

### 4.3 Effect of added materials on sulphur content in manure

The M+B manure contained a significantly less total S compared to the unenriched manure, whereas the total S value of the M+B+S variant was significantly the highest. The M+B manure was supplied with the biochar, i.e. the material with significantly lower S content compared to the unmatured manure, whereas the M+B+S was enriched by the excessive dose of elemental S together with biochar. The pyrolyzed matter has the potential to adsorb and stabilize any form of S transformation (Zhang et al., 2016; Lin et al., 2021) and mitigate its putative volatilization, e.g. in the form of H_2_S. Under insufficient aerobic conditions, one can expect a partial reduction of elemental S to H_2_S and its release into the environment.

### 4.4 Effect of added amendments on microbial abundance in manure

The co-fermentation of elemental sulphur and manure significantly modified biological properties; it led to the increased biomass of ammonia oxidizers and sulphur reducers. The addition of elemental S to the unmatured manure was crucial also for the abundance of microbiota. The significantly highest *dsr* value in the M+S was attributed to the absence of the putative biochar-mediated adsorption (as assumed for M+B+S) of elemental S, as described by Turk et al. (1992), which may function as a hindrance to the S reduction to H_2_S (Lovley and Phillips, 1994).

The abundance of ammonium oxidizers was significantly increased in the M+S and M+B+S variants compared to the unenriched manure and M+B; the highest AOB biomass was found in the M+B+S manure (**Figure 2B**). The obtained results may be explained by the increased availability of the substrate for the AOB-mediated oxidation (acidified ammonium $NH_{4}^{+}$ ) in the respective S-supplied manures, which the finding agreed with the previous observations (Gu et al., 2011; Soaud et al., 2011). The higher abundance of AOB in M+B+S was presumably due to a higher C_org_ and to general biochar-stimulated microbial growth.

The results of *nirS* determination (an indicator of denitrifying microflora in manure) were contrary to the AOB values: the lowest value in the M+B+S variant (significantly decreased compared to the control manure) and significantly the highest value in the M+B variant. Denitrification is the biochar-mediated and stimulated process that may occur simultaneously with nitrification (Cayuela et al., 2013). However, a low pH strongly interferes with the nitrate oxidation (Brenzinger et al., 2015), which present a considerable reason for the lower abundance of denitrifying microorganisms in the S-supplied variants.

### 4.5 Effect of manure types on soil fertility and plant biomass yield

The dry ABG was the key property for the evaluation of the agriculture benefit of the co-composted manure. Compared to the control, amendment of any type of manure to soil led to the significantly higher AGB. The highest AGB value was found in the M+B+S variant. The best fertilizing properties of M+B+S from all four used amendments were ascribed from the significantly highest content of dry biomass and total S in the respective manure. AGB correlated significantly (p ≤ 0.01) positively also with DHA (r = 0.66) and Ala-SIR (r = 0.62). The study by Yu et al. (2017) referred to a positive correlation between crop yield and soil N-NH_4_, the available P and K and microbial diversity or microbial abundance preservation. A beneficial increase of corn plant biomass in the soil amended with S-enriched biochar, which occurred due to the enhanced plant uptake of S (Zhang et al., 2016), corroborated the results with M+B+S manure.

All manure-amended variants increased the soil TC and TN content compared to the control. The TN values among all manure-amended variants were comparable. Albeit the non-enriched M manure was markedly C_org_-abundant; the soil M variant showed a significantly decreased TC compared to the M+B, M+S, M+B+S. However, both M+B+S and M+S manures showed higher ammonium nitrogen content and nitrification potential (AOB marker) compared to the M variant, and this indicated their higher N mineralization rate. Thus, enhanced C sequestration was presumably achieved due to application of manure with increased N conversion. The C:N ratio did not differ significantly between all variants. Both TC and TN correlated significantly (p ≤ 0.001) positively with DHA (r = 0.82 and 0.66, respectively), Ure (r = 0.77 and 0.66, respectively) and substrate-induced respirations, e.g. Tre-SIR (r = 0.64 and 0.55, respectively), NAG-SIR (r = 0.68 and 0.57, respectively), Lys-SIR (r = 0.67 and 0.54, respectively) and Arg-SIR (r = 0.76 and 0.66, respectively). These relations proved that the amendment of enriched manures (M+S, M+B+S, eventually M+B) enhanced microbial activity and mineralization due to the derived higher nutrient content and availability (Holatko et al., 2022).

### 4.6 Effect of manure types on soil microbial activity

The manures applied to the pot experiment with barley (*Hordeum vulgare* L.) led to differences in soil properties compared to the control unamended soil, and between the variants amended with unenriched manure and the enriched manures. All manure variants, applied to the soil, significantly enhanced DHA activity compared to the control (**Figure 3E**). The increase in DHA due to the combined effect of manure and biochar was already reported (Brtnicky et al., 2019; Yilmaz and Ergun, 2019). Furthermore, a significantly higher DHA value was obtained in the M+S variant compared to the M variant because the S-enriched manure exerted properties (lowest pH, highest N-NH_4_ and S-reducing microflora among the manures) that most enhanced the decomposing microbial activity in the amended soil. The higher access of S in the soil was reported to correlate with higher DHA (Katkar et al., 2011; Lemanowicz et al., 2020).

The elemental S amendment to manures (M+S, M+B+S) did not significantly change the soil ARS compared to the control, and the variants M and M+B showed even significantly lower ARS values. Our presumption of elemental S-stimulated enhancement of soil organic S mineralization (catalyzed by ARS), ascribed from (Castellano and Dick, 1991), was denied. On the contrary, the results imply the retarded S mineralization in the M and M+B soil variants due to the putatively higher portion of added mineral and readily available S, which might be caused by efficient ARS-mediated mineralization during the manure (M, M+B) fermentation. A similar significant biochar-derived increase in ARS was referred to in the soil environment (Khadem et al., 2019).

**Figure 6:**
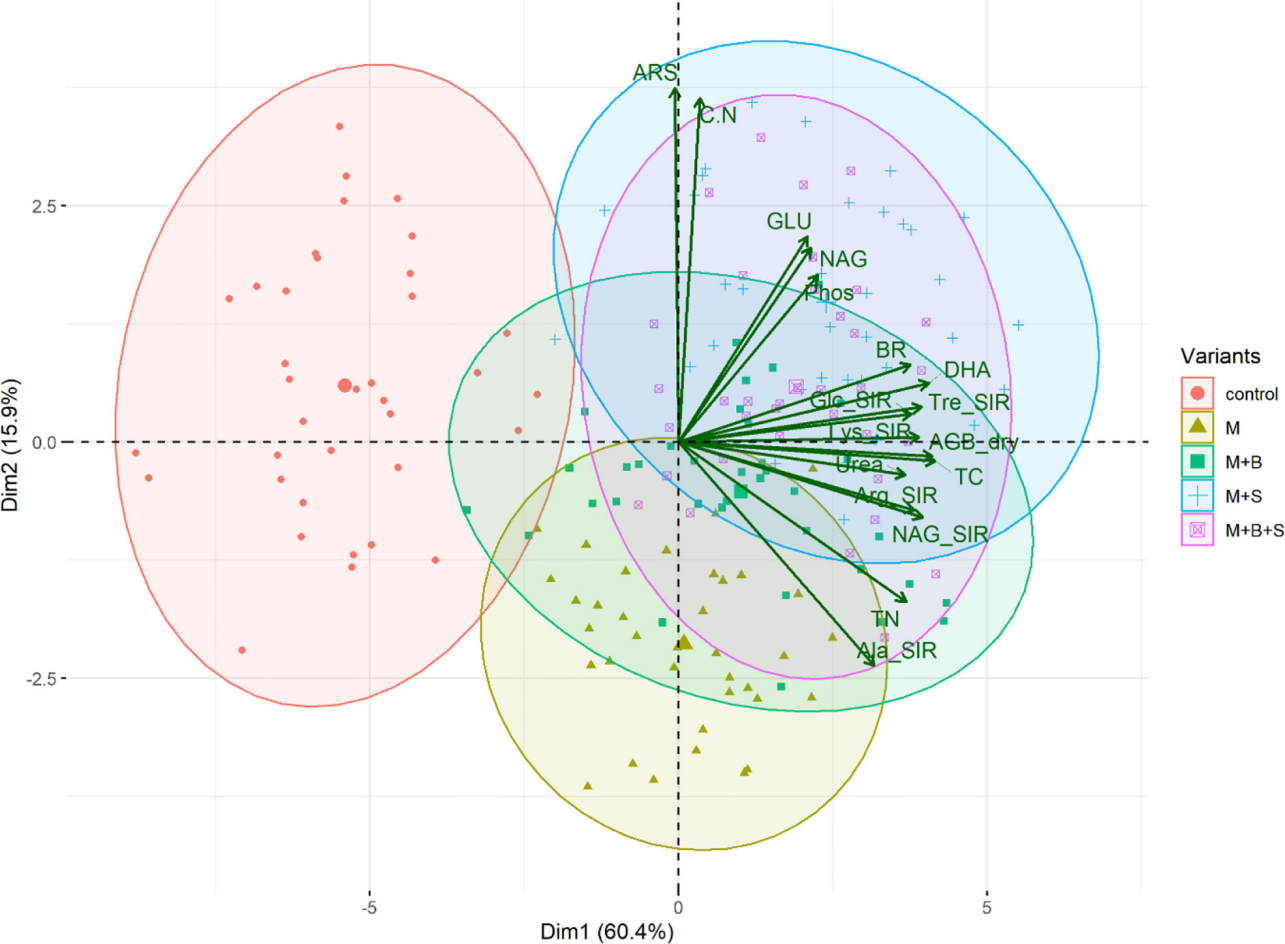
The PCA biplot of the soil properties.

Significantly increased Ure, Phos, NAG and GLU activities were observed in the variants M+S and M+B+S, compared to the control. The M+S variants also induced the Ure, NAG and GLU values compared to the M and M+B enzyme values. The Phos was the highest in M+B+S. Previously, elemental S amendment was referred to increase soil ARS, Phos and Ure (Godlewska, 2018a). A significant (p ≤ 0.001) positive correlation of Phos with Tre-SIR and Arg-SR (r = 0.66 and 0.59, respectively) and with dry AGB (r = 0.69) was found. NAG also correlated with AGB (r = 0.53; p ≤ 0.05). These relations implied that S-amendment mediated enhanced organic matter decomposition led to higher transformation and anticipated increased nutrient uptake for higher plant biomass yield. A similar benefit of combined use of biochar and poultry manure was referred to by Lu et al. (2015) to enhance microbial growth and enzyme activities (e.g. Ure). However, the significant beneficial synergic effect of elemental S and manure on the soil enzyme activities was novel and not yet mentioned in the literature. The enzyme activity dependence on SOM decomposition and their positive relation is known (Wutzler et al., 2017) and was shown in the PCA biplot (**Figure 6**).

**Figure 7:**
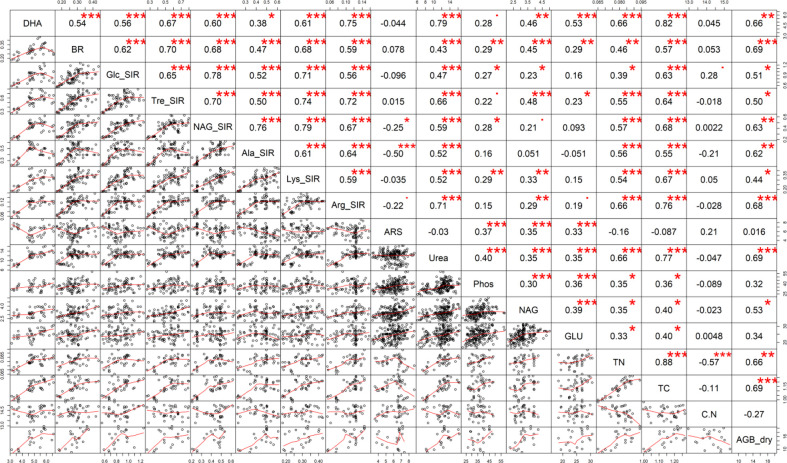
The Pearson’s correlation matrix of the soil properties. Explanation: Significance at · p ≤ 0.10; *p ≤ 0.05; **p ≤ 0.01; ***p ≤ 0.001.

S-enriched manure-treated variants (M+S and M+B+S) exerted higher basal respiration compared to both the control and the non-enriched manure variant M (**Figure 3K**). It verified our presumption of a significantly stimulating effect of S (co-composed with manure) on the enhancement of soil microbial abundance and activity. Enhanced microbial BR implied the intensified mineralization and putatively increased availability of nutrients for plants, which lead to higher plant biomass yield, TC and TN, as shown on the positive significant (p ≤ 0.001) correlation of BR and AGB (r = 0.69), TC (r = 0.57), and TN (r = 0.46, p ≤ 0.01) (**Figure 7**).

The results of all types of substrates-induced respirations seemed to be close to the results of BR, as shown by the significant (p ≤ 0.001-0.05) moderate to high correlation (r up to 0.79). Nevertheless, it was ascribed that manure enriched with biochar tended to stimulate more respiration inducible by N-rich substrates, whereas the application of S-enriched manure promoted higher respiration inducibility by the (non-nitrous) sugars. These differences implied a variable impact of used types of manure on functional soil diversity with the final consequence in the changes in the nutrient and other soil properties that affected the plant growth and biomass yield.

## 5 Conclusions

The sulphur-enriched manure showed the most lowered manure pH at the concurrent highest ammonium content. When manure, biochar and sulphur were co-fermented, the highest sulphur content and abundance of ammonium-oxidizing bacteria was observed. When added to soil, this biochar+sulphur-enriched manure promoted the highest dry aboveground plant biomass, the value was 98% higher compared to the unamended control, 38% higher compared to the amendment of biochar-enriched manure and 23% higher compared to the manure-amended variant. Sulphur-enriched manure types enhanced the most enzyme activities and soil respirations (basal, substrate-induced). Based on the results obtained, it was concluded that the co-fermentation of biological manures with bio-based materials, such as biochar and sulphur resulting as a by-product of biogas, is an attractive approach, not only to improve the enriched manure product but also to enhance soil fertility, health and crop productivity. This improvement of organic fertilizers may contribute to the circular economy and it will be further investigated by up-scaling on the field level.

## Data availability statement

The original contributions presented in the study are included in the article/supplementary material. Further inquiries can be directed to the corresponding authors.

## Author contributions

Conceptualization, MB and TH; methodology, TH, AK, and OL; software, TB; validation, TB, PS, PR, and JH; formal analysis, TH; investigation, AM; resources, JH and OL; data curation, TH, OL, and AK; writing - original draft preparation, JH, TH, AM, and PS; writing - review and editing, TH, AK, AM, MN, PS, PR, and MB; visualization, TB and AM; supervision, MB, TH, and MN; project administration, MB and AK.; funding acquisition, JH, AK, and MB. All authors have read and agreed to the published version of the manuscript.

## Funding

The work was supported by the project of Technology Agency of the Czech Republic number TH04030142, by the Ministry of Agriculture of the Czech Republic, institutional support MZE-RO1218 and MZE-RO1722 and by Ministry of Education, Youth and Sports of the Czech Republic, grant number FCH-S-22-8001.

## Conflict of interest

The authors JH, OL and AK are employed by Agrovyzkum Rapotin, Ltd., Vyzkumniku 267, 788 13 Rapotin, Czech Republic and Agricultural Research, Ltd., Troubsko, Czech Republic.

The remaining authors declare that the research was conducted in the absence of any commercial or financial relationships that could be construed as a potential conflict of interest.

## Publisher’s note

All claims expressed in this article are solely those of the authors and do not necessarily represent those of their affiliated organizations, or those of the publisher, the editors and the reviewers. Any product that may be evaluated in this article, or claim that may be made by its manufacturer, is not guaranteed or endorsed by the publisher.
